# Multi-Mycotoxin Screening Reveals the Occurrence of 139 Different Secondary Metabolites in Feed and Feed Ingredients

**DOI:** 10.3390/toxins5030504

**Published:** 2013-03-08

**Authors:** Elisabeth Streit, Christina Schwab, Michael Sulyok, Karin Naehrer, Rudolf Krska, Gerd Schatzmayr

**Affiliations:** 1 BIOMIN Research Center, Technopark 1, Tulln 3430, Austria; E-Mails: elisabeth.streit@biomin.net (E.S.); gerd.schatzmayr@biomin.net (G.S.); 2 BIOMIN Holding GmbH, Industriestrasse 21, Herzogenburg 3130, Austria; E-Mail: karin.naehrer@biomin.net; 3 Center for Analytical Chemistry, Department for Agrobiotechnology (IFA-Tulln), University of Natural Resources and Life Sciences, Vienna (BOKU), Konrad-Lorenz-Str. 20, Tulln 3430, Austria; E-Mails: michael.sulyok@boku.ac.at (M.S.); rudolf.krska@boku.ac.at (R.K.)

**Keywords:** emerging mycotoxins, masked mycotoxins, liquid chromatography, mass spectrometry, LC-MS/MS, mould

## Abstract

The development of liquid chromatography-mass spectrometry (LC-MS)/mass spectrometry (MS) methods for the simultaneous detection and quantification of a broad spectrum of mycotoxins has facilitated the screening of a larger number of samples for contamination with a wide array of less well-known “emerging” mycotoxins and other metabolites. In this study, 83 samples of feed and feed raw materials were analysed. All of them were found to contain seven to 69 metabolites. The total number of detected metabolites amounts to 139. *Fusarium* mycotoxins were most common, but a number of *Alternaria* toxins also occurred very often. Furthermore, two so-called masked mycotoxins (*i.e.*, mycotoxin conjugates), namely deoxynivalenol-3-glucoside (75% positives) and zearalenone-4-sulfate (49% positives), were frequently detected. Although the observed median concentrations of the individual analytes were generally in the low μg/kg range, evaluating the toxicological potential of a given sample is difficult. Toxicity data on less well-known mycotoxins and other detected metabolites are notoriously scarce, as an overview on the available information on the most commonly detected metabolites shows. Besides, the possible synergistic effects of co-occurring substances have to be considered.

## 1. Introduction

Mycotoxins are a chemically diverse group of toxic secondary fungal metabolites that can occur in a wide array of commodities. Strictly speaking, they are defined as secondary metabolites of fungal origin exhibiting *in vivo* toxicity towards vertebrates after introduction via a natural route (*i.e.*, ingestion, inhalation *etc*.) [1]. Contamination results from fungal infection and proliferation in the field or during storage [2,3]. Legal authorities, food and feed industry alike acknowledge the importance of this issue and considerable effort is directed towards detecting and preventing mycotoxin contamination. However, of the 300–400 mycotoxins known to date [4], only a very limited number is subject to legal guidance and regular monitoring. Regarding feed, aflatoxins (AF), fumonisins (FB), deoxynivalenol (DON), zearalenone (ZEN) and ochratoxin A (OTA) are most often tested for [2]. Still, many analytical methods used for the determination of fungal metabolites are specific for one mycotoxin or a closely related group of mycotoxins. Therefore, a separate analysis is required for each mycotoxin of interest, an important constraint for monitoring a large number of mycotoxins. The development of multi-mycotoxin LC-MS/MS methods was a major step towards overcoming this problem. A very powerful method was established by Sulyok *et al.* in 2006 [5] and has been continuously extended ever since [6,7]. The parameters for the detection of 186 metabolites have been published by Vishwanath *et al.* [8], and the same group has expanded the method to cover 320 fungal, bacterial and plant metabolites since then.

In addition to providing information on occurrence and concentration levels of an increasing number of usually neglected mycotoxins multi-mycotoxin LC-MS/MS methods also help to address the issue of masked mycotoxins. Masked mycotoxins are mycotoxin conjugates that typically remain undetected when testing for the parent toxin. They may be formed as part of the plant’s defence mechanism (e.g., DON-3-glucoside or ZEN-4-glucoside), and in some cases, e.g., hidden fumonisins, masked mycotoxins even develop during food processing [9]. Apart from exerting toxic effects of their own there is evidence suggesting that some masked mycotoxins are reconverted into the parent toxin during digestion, further adding to the toxicity of the respective food or feed [10,11,12,13].

Ever since 2005, BIOMIN has conducted a survey program monitoring the worldwide prevalence and concentration of aflatoxins, fumonisins, deoxynivalenol, zearalenone and ochratoxin A in feed and feed raw materials [14,15,16,17,18,19,20]. Additionally, in an effort to further broaden the knowledge on mycotoxin occurrence and co-occurrence in feed, 83 samples were subjected to multi-mycotoxin LC-MS/MS analysis. The results are presented in this paper, including an overview of the currently available toxicity data for the most commonly detected “novel” or “emerging” mycotoxins (*i.e.*, mycotoxins that have not received as much scientific attention as AF, FB, DON, ZEN and OTA).

## 2. Results

**Figure 1:**
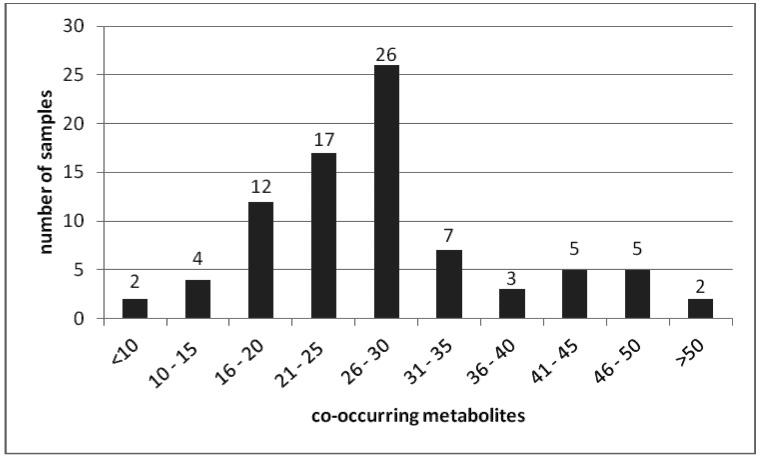
Number of samples co-contaminated with a given number of mycotoxins and/or metabolites.

**Figure 2:**
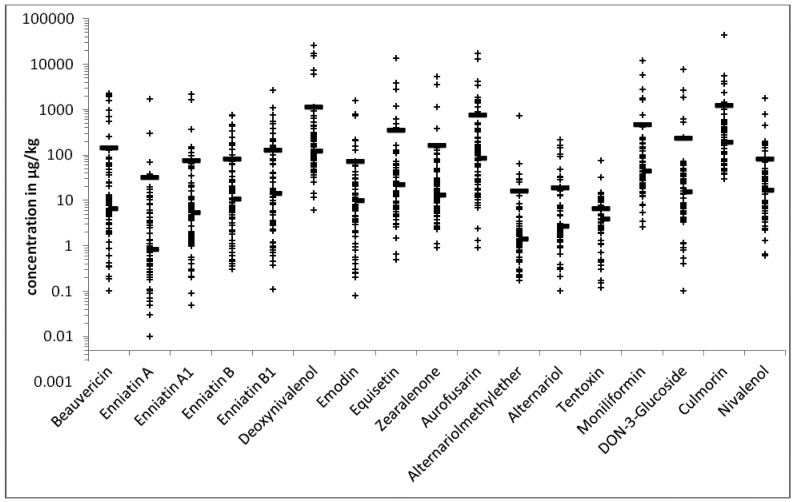
Distribution of the concentrations of metabolites detected in more than 60% of the samples; median (right side line, ־) and average (continuous line, ─) concentration are given as well.

A total number of 139 different fungal metabolites were detected in the 83 feed samples (Table 1). All of the samples were co-contaminated with seven to 69 different mycotoxins (Figure 1). The co-occurrence of 28 metabolites was most frequent, observed in *n* = 9 of the samples. Figure 2 shows the distribution of the detected concentrations for metabolites that were detected in more than 60% of the samples. 

**Table 1:** Mycotoxins and metabolites detected in the liquid chromatography-mass spectrometry (LC-MS)/ mass spectrometry (MS) analysed samples, specifying the number of positive samples (*n* (pos)), the percentage of positive samples, as well as the median (positives) and the maximum concentration in μg/kg. In case there were only two positives, the range is provided instead; analytes are listed in order of prevalence.

Mycotoxin/metabolite		*n* (pos)	% pos	Median (μg/kg)	Max (μg/kg)	Mycotoxin/metabolite		*n* (pos)	% pos	Median (μg/kg)	Max (μg/kg)
Beauvericin		81	98	6.7	2326	Pestalotin		5	6	10	19
sum of Enniatins		80	96	30	5441	Tetracycline (ab)		5	6	77	10,696
	Enniatin A1	79	95	5.5	2216	Versicolorin C **		5	6	0.8	89
	Enniatin B	76	92	11	780	Amoxycillin * (ab)		4	5	NA	NA
	Enniatin B1	76	92	14	2690	Andrastin D *		4	5	NA	NA
	Enniatin A	72	87	0.8	1745	Dechlorogriseofulvin		4	5	18	182
	Enniatin B2	8	10	0.8	13	Griseofulvin		4	5	31	399
	Enniatin B3	7	8	0.01	0.1						
Deoxynivalenol		74	89	122	25,928	Linamarin (plt)		4	5	1705	20,205
Emodin		74	89	9.8	1570	Monoacetoxyscirpenol		4	5	7.7	31
Equisetin		72	87	23	13,680	Neoxaline		4	5	3.3	13
Zearalenone		72	87	14	5326	Penitrem A		4	5	92	701
Aurofusarin		70	84	85	17,659	Roquefortine C		4	5	103	915
Alternariol methyl ether		68	82	1.4	733	Secalonic acid D		4	5	4.5	369
Alternariol		66	80	2.8	221	3-AcetylDON		3	4	28	588
Tentoxin		66	80	3.9	76	Agroclavin		3	4	0.1	0.9
Moniliformin		63	76	45	12,236	Aminodecyl octadecanol		3	4	39	276
DON-3-glucoside		62	75	15	7764	Cytochalasin J		3	4	27	164
Culmorin		61	63	195	44,616	Ergocorninine		3	4	14	15
Nivalenol		61	63	17	1760	Ergocristine		3	4	47	63
Tryptophol		59	71	267	99,040	Ergocristinin		3	4	21	25
Apicidin		55	66	1.9	160	Ergocryptine		3	4	16	25
Brevianamide F		54	65	69	2043	Ergocryptinine		3	4	8.8	11
Tenuazonic acid		54	65	68	1983	Ergosin		3	4	27	52
15-Hydroxyculmorin		52	63	49	15,620	Ergosinin		3	4	4.8	8.6
Butenolide		43	52	23	1490	Ergotamine		3	4	71	129
ZEN-4-sulfate		41	49	1	136	Ergotaminine		3	4	9.7	18
Altertoxin-I		35	42	1.1	65	Lotaustralin (plt)		3	4	50	435
Curvularin		29	35	14	484	Ochratoxin A		3	4	4.9	31
Avenacin Y		26	31	209	9948	Paspalitrem A *		3	4	NA	NA
Macrosporin A		26	31	3.3	9.1	Rubellin D		3	4	259	1188
T-2 Toxin		26	31	3.8	427	Rubrofusarin		3	4	2374	4923
Macrosporin		24	29	3.7	15	T-2 Tetraol		3	4	9.5	1655
Monocerin		24	29	0.9	2644	sum of Aflatoxins		2	2	0.24 ^a^	861
Siccanol *		23	28	9607	39850		Aflatoxin B_1_	2	2	0.24 ^a^	699
3-Nitropropionic acid		19	23	6	392		Aflatoxin B_2_	1	1	-	63
sum of Fumonisins		18	22	203	57,667		Aflatoxin G_1_	1	1	-	69
	Fumonisin B_1_	18	22	142	40,300		Aflatoxin G_2_	1	1	-	4.4
	Fumonisin B_2_	18	22	38	10,372		Aflatoxin M_1_	1	1	-	26
	Fumonisin B_3_	14	17	18	3859	Cyclopiazonic acid		2	2	37 ^a^	2319
	Fumonisin B_4_	7	8	112	3136	Diacetoxyscirpenol		2	2	0.04 ^a^	2.7
Fusaric acid		18	22	643	13,593	Ergocornine		2	2	19 ^a^	25
HT-2 Toxin		18	22	13	1910	Ergocristam *		2	2	17,370 ^a^	19230
Chrysophanol		17	20	8.2	41	Ergocristinam *		2	2	7181 ^a^	8233
Mycophenolic acid		17	20	40	21,856	Ergovalin		2	2	1 ^a^	5.4
Averufin **		16	19	0.4	215	Gibberellic acid		2	2	9.7 ^a^	89
β-Zearalenol		15	18	5.1	174	Methylsterigmatocystin		2	2	0.4 ^a^	27
Bikaverin *		14	17	27510	51,130	Neosolaniol		2	2	0.7 ^a^	290
Fusaproliferin		14	17	2555	14,844	Penicillic acid		2	2	12 ^a^	13
Fusarinolic acid **		13	16	643	13,965	Radicicol		2	2	5.3 ^a^	5.9
Skyrin		13	16	1.4	6853	Terphenyllin		2	2	16 ^a^	67
Chlamydosporol		12	14	55	656	15-AcetylDON		1	1	-	2718
5-Hydroxyculmorin		11	13	350	3920	Aflatrem *		1	1	NA	NA
α-Zearalenol		11	13	2	51	Averantin		1	1	-	20
Ergometrinin		11	13	0.3	14	Averufanin **		1	1	-	29
Kojic acid		10	12	75	3172	Calphostin C		1	1	-	431
Chanoclavin		8	10	0.1	8.9	Citrinin		1	1	-	42
Andrastin A *		7	8	4814	7603	Cycloaspeptide A		1	1	-	4.6
Physcion		7	8	245	1162	Cytochalasin B		1	1	-	27
Sterigmatocystin		7	8	1.5	4.7	Cytochalasin H		1	1	-	77
15-Hydroxyculmoron		6	7	83	4886	Decalonectrin		1	1	NA	NA
Aspterric acid		6	7	194	3992	Erginie		1	1	-	0.33
hydrolysed FB_1_		6	7	15	95	Festucalvin		1	1	-	0.34
Paspalin *		6	7	NA	NA	Malformin A **		1	1	-	8
Sambucinol		6	7	149	675	Malformin C		1	1	-	2.7
Citreoviridin		5	6	94	382	Nidurufin **		1	1	-	9
Ergometrin		5	6	6.8	26	Norsolorinic acid **		1	1	-	1.6
Lycomycin *		5	6	NA	NA	Pyrenophorol		1	1	-	13
Meleagrin		5	6	237	1821	Rugulosin		1	1	-	201,640
Oxytetracyclin		5	6	NA	NA	T-2 Triol		1	1	-	278
Paxillin		5	6	5017	94,860	Versicolorin A **		1	1	-	27

^a^ minimum in µg/kg; * no standard available for quantification (if a standard became available at some point, the results samples analysed thereafter are shown in the median/maximum column); ** semi-quantified using response factor of a structurally related analyte; ab: antibiotic, plt: plant metabolite, NA: not applicable.

Beauvericin (BEA) was found most often. It was detected in 98% of the samples. Enniatins (ENN) were second most common, with 96% of the samples testing positive followed by DON and emodin with 89% positives each. 

The samples listed in Table 1 include six maize cob samples from Lower Austria that all had visible signs of mould infection, ranging from slightly infected (*n* = 4) to severely moulded (*n* = 2). Two of those samples were obtained in 2010 and the other four in 2012. All samples were acquired directly after harvest. The results per sample are specified in Table 2.

**Table 2:** Analytes detected in six maize cob samples from Lower Austria, showing different degrees of mould infection.

Mycotoxin/metabolite	Samples, concentrations in μg/kg					
	2010-1 *	2010-2	2012-1	2012-2	2012-3	2012-4 *
15-acetyl-DON	-	-	-	-	-	2718
15-Hydroxyculmorin	-	-	-	-	-	4634
3-acetyl-DON	-	-	-	-	-	588
α-Zearalenol	-	-	-	-	-	35
Apicidin	0.03	22	2	-	-	-
Aurofusarin	240	3380	1.3	44	0.9	17,659
Avenacin Y	1210	-	-	-	-	-
Beauvericin	1390	970	1591	64	699	57
β-Zearalenol	-	-	-	-	-	174
Bikaverin	-	-	detected	detected	detected	detected
Butenolide	167	1490	32	167	-	569
Culmorin	-	-	-	-	-	44,616
Decalonectrin	-	-	-	-	-	detected
Deoxynivalenol	12	43	65	48	36	25,928
Diacetoxyscirpenol	0.04	2.7	-	-	-	-
DON-3-glucoside	-	0.5	0.1	-	0.1	7764
Enniatin A	301	0.6	-	8.3	-	-
Enniatin A1	1670	4.9	0.4	83	0.3	0.5
Enniatin B	780	3.9	0.6	64	0.3	0.3
Enniatin B1	2690	9.8	1	166	0.7	0.8
Equisetin	-	-	-	-	4.4	1.5
Fusaproliferin	1970	46	14,844	975	5029	-
Fusaric acid	-	-	2146	1155	1426	85
Fusarinolic acid	-	-	9405	440	3960	1936
Gibberellic acid	-	-	9.7	-	-	-
Moniliformin	5750	1650	2809	244	1,779	231
Monoacetoxyscirpenol	0.64	6.1	-	-	-	-
Nivalenol	147	1760	2.3	-	-	41
Pestalotin	-	-	2.2	19	10	-
Sambucinol	-	-	-	-	-	181
Siccanol	-	-	detected	detected	detected	detected
Skyrin	-	10	-	-	-	-
Tentoxin	-	-	0.5	2.8	-	-
Tenuazonic acid	-	-	499	134	-	133
Zearalenone	-	-	-	-	-	5326
ZEN-4-sulfate	-	-	-	-	-	136

* Heavily moulded.

The six maize cob samples were co-contaminated with 15 to 25 metabolites, which are below the average of 28 co-occurring metabolites observed in the pool of samples as a whole. Great differences were observed regarding the mycotoxin concentrations and contamination pattern depending on the year of analysis. Mono- and di-acetoxyscirpenol for example were only detected in the 2010 samples, while tentoxin and tenuazonic acid were only detected in 2012 (bikaverin, fusaric acid, fusarinolic acid and siccanol were not covered by the 2010 method). Nivalenol levels were higher in 2010, while fusaproliferin concentrations were more elevated in 2012 (sample 2010-2 had the highest nivalenol concentration, and sample 2012-1 had the highest fusaproliferin concentration of all 83 samples). It is interesting to note that the mycotoxin levels and contamination pattern were quite different for samples sourced in the same year as well in some cases. The enniatin concentrations in the heavily moulded cob from 2010 (2010-1) were many times higher than those detected in the other samples. This sample also contained higher levels of fusaproliferin or moniliformin than the other 2010 sample, but had much lower levels of aurofusarin, mono- and di-acetoxyscirpenol or nivalenol. Sample 2012-4 (severe mould infection), for example, contained much higher levels of aurofusarin, DON and DON-3-glucoside than any of the other samples and was the only sample contaminated with 3-acetyl-DON, 15-acetyl-DON, 15-hydroxyculmorin, α-zearalenol, β-zearalenol, culmorin, ZEN or ZEN-4-sulfate. In fact, the observed concentrations of DON, ZEN, aurofusarin, DON-3-glucoside, ZEN-4-sulfate and 3-acetyl-DON in this sample were the highest in the entire study. On the other hand, it did not contain fusaproliferin, which was present at rather high concentrations in the other three samples from 2012. These results suggest that the fungal populations on a given year’s cobs were different, despite the fact that the cobs were obtained from the same field. 

Furthermore, the pool of samples includes three US maize samples that were selected for LC-MS/MS analysis after “conventional” high-performance liquid chromatography (HPLC) analysis showed a high contamination with DON (*n* = 2) or fumonisins and aflatoxins (*n* = 1). This was done in order to identify co-occurring metabolites in samples with excessively high concentrations of regulated mycotoxins. Both samples with high DON levels (17,360 and 15,684 μg/kg) also had high levels of DON-3-glucoside (2696 and 1977 µg/kg), aurofusarin (4155 and 12,908 µg/kg), ZEN (1137 and 378 μg/kg) and ZEN-4-sulfate (61 and 26 μg/kg). Fumonisin levels were low in both samples (sum of fumonisins: 35 and 56 μg/kg). The latter sample also had the highest concentration of 15-hydroxyculmorin detected in this study. The samples were co-contaminated with 11 and 45 metabolites, respectively. The aflatoxin and fumonisin contaminated sample tested positive for 69 metabolites. Apart from containing the highest aflatoxin and fumonisin levels observed in this study, it also had the highest level of cyclopiazonic acid, moniliformin and fusarinolic acid. Furthermore, 49 μg/kg alternariol, 39 μg/kg alternariol methyl ether and 30 μg/kg OTA were detected amongst others. DON and ZEN concentrations were low in this sample (79 and 3 μg/kg, respectively).

## 3. Discussion

The results of the analysis of 83 samples with a multi-mycotoxin method based on LC-MS/MS clearly show that mycotoxin co-contamination is the rule. Mixtures of *Fusarium* toxins are detected most frequently, which is in line with the findings of two other studies using the same LC-MS/MS method on African food and feed samples [21,22]. Given the fact that *Fusarium* species are plant pathogens of major importance worldwide, particularly in cereal production, this is not surprising. Their occurrence in cereals and feed on a global scale was reviewed by Placinta *et al.* [23]. Bottalico [24] published an extensive review on the occurrence of various *Fusarium* spp. in Europe, including their mycotoxin profiles. Most species were reported to simultaneously produce a number of mycotoxins. Additionally, crops are almost always infected by several different fungal species at once. Bottalico [24] stated that it was quite common to isolate up to nine different *Fusarium* spp. from a single piece of infected tissue. Taken together, these facts explain the high prevalence of *Fusarium* mycotoxins in the analysed samples. 

Except for specifically selected high contamination samples, the concentrations of individual mycotoxins were generally low and almost always well below the respective EC maximum level or guidance value (maximum levels exist for aflatoxin B1 (AFB_1_) [25], guidance values exist for DON, ZEN, FB1 + FB2 and OTA [26]) in feed. As for DON, the frequent detection of DON-3-glucoside (DON-3-Glc) should be mentioned. It always co-occurred with DON and was detected in 75% of the samples. Given that several lactic acid bacteria commonly found in the digestive tract of mammals were recently reported capable of cleaving a significant proportion of DON-3-Glc, releasing DON [12], the presence of DON-3-Glc is likely to increase the total bio available amount of DON in contaminated feed. In this study, DON-3-Glc was detected at concentrations attaining on average 12% of the DON contamination level of the respective sample. This percentage is in line with the findings of De Boevre *et al.* [27], who reported that DON-3-Glc accounted for up to 14% of the average total DON equivalents detected in feed samples. Nevertheless, even supposing the unlikely event of a 100% conversion of DON-3-Glc to DON during digestion and disregarding specifically selected high contamination samples, only two wheat samples came close to surpassing the respective European Commission (EC) guidance value. Recent results obtained from rat studies suggest, however, that the bioavailability of DON-3-Glc is low [28]. ZEN-4-sulfate (ZEN-4-S), a masked form of ZEN, was also detected frequently. This mycotoxin conjugate is readily hydrolysed in acidic conditions [11]. Hence, ZEN is released during digestion of ZEN-4-S contaminated diets. 

Concerning the mycotoxins other than DON and ZEN listed in Table 1, data on toxicity, especially *in vivo* data, are scarce. Available data are summarised in Table 3. BEA and ENN exhibit low acute toxicity *in vivo* [29]. It is, however, unclear to what extent they influence the toxic effects of other mycotoxins. BEA in particular was reported to be a chemo sensitising agent with the potential to increase the efficacy of antibiotics, as well as chemotherapeutic anti-cancer drugs [29]. 

Emodin has also been investigated for its pharmacologic properties. It is a major chemical constituent of Chinese rhubarb (*Rheum palmatum*) roots and has been reported to exert anti-inflammatory, anti-arteriosclerotic and immunosuppressive effects. Furthermore, it inhibits the proliferation of various cancer cell lines [30], increases the effect of gemcitabine on pancreatic cancer in mice [31] and has been proposed as possible treatment for type-2 diabetes [32]. 

**Table 3:** Producing species, effects and toxicities of less well-known mycotoxins and other metabolites detected in more than 45% of the samples.

Metabolite	Produced by	Effects
**Alternariol**	*Alternaria* sp. *A. alternata* *A. triticina* *A. arborescens*	conflicting results regarding mutagenic activity (strongly mutagenic in *Bacillus subtilis* rec assay and *E. coli* ND 160 reverse mutation assay; weak to no mutagenic activity in Ames test) genotoxic (causes DNA strand breaks by interacting with mammalian topoisomerases); implicated as risk factor for oesophageal cancer (reviewed in [33]) **chicken embryos**: no mortality or teratogenic effects in chicken embryos at doses up to 1000 mg/egg [34]
**Alternariol methyl ether**	*Alternaria alternata,* *A. triticina* *A. cucumerina* *A. dauci* *A. kikuchiana* *A. solani* *A. arborescens*	genotoxic (causes DNA strand breaks by interacting with mammalian topoisomerase IIα) (reviewed in [33]); brain haemorrhages and bleeding in cerebral ventricles; effect on progesterone synthesis in pigs; therefore, postulated effect on reproductive performance in pig and other mammalian species [35] **mice**: precancerous changes in oesophageal mucosa of mice fed 100 mg/kg bw/day for 10 months, also causing weight loss, lower food consumption (less toxic effect than tenuazonic acid (25 mg/kg bw/day for 10 months)) [36] **chicken embryos**: no mortality or teratogenic effects in chicken embryos at doses up to 500 mg/egg; [34]
**Apicidin**	*Fusarium* sp. *F. pallidoreseum (F. semitectum)*	histone deacetylase inhibitor, broad spectrum antiprotozoal activity [37]; antiproliferative [38] and apoptotic [39] in various cancer cell lines **brine shrimp larvae**: LD_50_: 40 mg/mL; [40] **rats**: died with 0.05% apicidin supplementation of diet; apicidin causes body weight loss, haemorrhage in the stomach, intestines and bladder [40]
**Aurofusarin**	*F. graminearum* *F. acuminatum* *F. avenaceum* *F. crookwellens* *F. culmorum* *F. graminearum* *F. poae* *F. sambucinum* *F. tricinctum*	major effect on poultry are changes in egg yolk colour from yellow-orange to dark-brown [41,42]; decrease of protein and fat content in chicken meat [43] **quail eggs**: 26.4 mg/kg feed influenced quality of eggs—decrease in vitamins E, A, total carotenoid, lutein and zeaxanthin concentrations and significantly increased egg yolk susceptibility to lipid peroxidation [44] **breeding chickens**: 26.4 mg/kg feed compromised the immune system of the progeny [45]
**Beauvericin**	*Beaveria bassiana* *F. moniliforme* *F. avenaceum* *F. subglutianans* *F. proliferatum* *F. tricinctum* *F. poae* [46,47]	antibacterial, insecticidal, cytotoxic, (reviewed in [29,48]); ionophoric properties cause dysfunction of mitochondria [49] and induces apoptosis (reviewed in [29,48]); co-occurs with enniatin A [50]; bioactivities are different to enniatins due to 3 *N*-methylphenylalanyl residues [48] **acute toxicity** (summarised in [29]) **mice**: LD_50_: ≥10 mg/kg bw, intraperitoneal; ≥100 mg/kg bw oral **low/no acute toxicity in**: **duckling** (100 mg/kg bw, gastric intubation) **turkey** (2.5 mg/kg feed) **broiler** (12 mg/kg feed)
**Brevianamide F**	*Penicillium brevicompactum* *Aspergillus versicolor* *A. fumigatus*	antibacterial, [51] precursor of the fumitremorgins and the tryprostatins [51,52] also produced by *Streptomyces* sp. [53]
**Butenolide**	*Fusarium crookwellense* *F. culmorum* *F. graminearum* *F. poae* *F. moniliformin*	myocardial oxidative damage; [54] oxidative injuries in chick embryonic livers and kidneys [55]; growth retardation and differentiation inhibition *in vitro* in rat embryos [56]; probably implicated in fescue foot disease [57]; anti-marine-fouling compound—toxic to zebra fish embryos by inducing apoptosis [58] **mice**: LD_50_: 44 mg/kg bw, intraperitoneal; 275 mg/kg bw oral [59] **steers**: died after 68 and 39 mg/kg for 2 and 3 days oral dosage; with smaller dosage, development of petechial haemorrhages and acute inflammation of gastric compartments ([60] in [59])
**Culmorin**	*F. culmorum,* *F. graminearum* *F. poae* *F. langsethiae* *F. cerealis*	low toxicity in several *in vitro* assays, Ames test negative [61], co-occurrence with DON [62] **swine**: some minor non-significant effects on growth and feed consumption (2 mg/kg feed) with and without DON (6 mg/kg feed) [63]
**15-Hydroxy-culmorin**	*F. graminearum* *F. culmorum* *F. poae*	co-occurrence with culmorin and DON [64] no evidence of acute toxicity to mammals so far [65]
**Emodin**	*A. wentii* *A. flavus* *A. ocharaceus* *Plants:rhubarb root*	possibly genotoxic [66]; cytotoxic, antitumour, antibacterial, anti-inflammatory, immunosuppressive [30] active ingredient of various Chinese herbs, potential drug for therapy of type-2 diabetes [32] **mice**: induces embryonic toxicity in mouse blastocysts through apoptosis; [30] **1-day-old cockerels**: LD_50_: 3.7 mg/kg bw [67] clinical symptoms in cockerels included loss of appetite, accumulation of faecal material with acute epidermal irritation around the cloaca, general debilitation and mortality within 5 days of ingestion; **zebrafish embryos**: 0.25 μg/mL negatively affected embryo survival and hatching success; toxic to larvae at relatively low concentrations [68]
**Enniatin A** **Enniatin A1** **Enniatin B** **Enniatin B1**	*F. moniliforme* *F. avenaceum* *F. roseum* *F. solani* *F. nivale* *F. acuminatum*	antibacterial, antifungal, herbicidal, insecticidal, ionophore, induction of apoptosis [29,49,69,70] enniatin B often occurs together with enniatin B1 and enniatin A with beauvericin [50] **mice**: 10–40 mg/kg bw, intraperitoneal, every 8 h: mice died within 2–5 days; doses <10 mg/kg bw resulted in reduced body weight [71] **brine shrimp**: 50 μg enniatin B/mL killed almost all after 24 h; ranking in brine shrimps: enniatin B > B1 > A1 > A, mixture of all four most toxic [72]
**Equisetin**	*F. equiseti* *F. semitectum*	antibiotic activity against Gram-positive bacteria [73]; potent inhibitor of HIV-1 integrase enzyme [74] **mice**: LD_50_: 63.0 mg/kg bw, intraperitoneal [73]
**Moniliformin**	*F. avenaceum* *F. subglutinans* *F. proliferatum* *F. oxysporum* *F. fusariodes*	**1-day-old chicks**: LD_50_: 5.4 mg/kg bw oral [75] **7-weeks-old broilers**: LD_50_: 1.38 mg/kg bw, intravenous [76] **broilers**: 50 mg/kg feed toxic for broilers fed to market age, resulting in lower body weight gain, less efficient feed converting rate, higher mortality [77]; additive effects with aflatoxin [78], as well as with deoxynivalenol [79] **turkeys**: 37.5 mg/kg feed hepatotoxic; 25 mg/kg feed cardiotoxic [77] **swine**:100 mg/kg feed for 28 days in growing barrows reduced body weight, body weight gain and feed consumption and affected serum biochemical analytes; 50 mg/kg feed influence on haematologic values [80]; additive effects with fumonisin B_1_[81] **sheep**: 10 mg/kg bw, intubation caused death after 18 h, also degeneration of the proximal tubules of kidneys [82]
**Tentoxin**	*Alternaria alternata*	induces chlorosis in germinating seedlings of many dicotyledonous plants [64]
**Tenuazonic acid**	*Alternaria* sp. *A. alternata* *A. triticina* *A. tenuis* *A. arborescens*	**mice**: LD_50_: 81 mg/kg bw oral, female; 186 mg/kg bw oral, male [83]; risk for oesophageal cancer: toxic effect on oesophageal mucosa of mice (25 mg/kg bw/day oral for 10 months) stronger than with alternariol methyl ether (100 mg/kg bw/day oral for 10 months)—resulting in weight loss and lower feed consumption [36] **rats**: LD_50_: 168 mg/kg bw oral, female; 180 mg/kg bw oral, male [83] **young chicken**: LD_50_: 37.5 mg/kg bw oral [84] 1.25–1.50 mg/kg bw/day (3-weeks oral) induced microscopic and macroscopic lesions in various tissues and significant weight loss [84]
**Tryptophol**	*Candida albicans* *Acremonium lolii*	*in vitro*: cytotoxic, cytostatic, genotoxic effects in lymphocytes [85]; 2 mM (24h) damages DNA in HepG2, A549 and THP-1 cells (comet-assay) [86]; induction of apoptosis in leukaemic blood monocytes cell line U937 [87] **mice**: LD_50_: 351 mg/kg bw, intraperitoneal [88]

As for the *Alternaria* metabolites alternariol, alternariol methyl ether, tenuazonic acid and tentoxin, only the first three are of toxicological concern. Tentoxin exhibits herbicidal properties [64,89], as it induces chlorosis by inhibiting chloroplast development [64]. It does not even figure in some reviews on *Alternaria* mycotoxins (e.g., Scott [90]) or is mentioned in passing as co-occurring with more important toxins, such as alternariol or alternariol methyl ether [33,91]. Alternariol and alternariol methyl ether have received some attention, due to their mutagenic and genotoxic properties. Their acute toxicity is rather low [33,90,91]. Tenuazonic acid is considered the most acutely toxic of the *Alternaria* mycotoxins [92]. Exposure to sub-lethal levels of tenuazonic acid (10 mg/kg) negatively affects feed efficiency in chicken and causes lesions on various organs [84]. The occurrence of *Alternaria* mycotoxins in food and feed in general was not deemed a significant health hazard in a report submitted to the European Food Safety Authority (EFSA), owing to the low naturally occurring contamination levels [93]. The EFSA panel on contaminants in the food chain (CONTAM) stated that a risk assessment for *Alternaria* mycotoxins in feed was not possible, due to a lack of information on occurrence and toxicity [94]. 

Data on culmorin and its hydroxylated analogues do not indicate acute toxicity to mammals [61]. Moniliformin (MON) *in vivo* toxicity has been investigated more extensively. Poultry seem to be the most sensitive species. The main acute symptoms are muscular weakness, respiratory stress and myocardial degeneration [29], and chronic exposure will result in reduced weight gain, impaired immune system and myocardial lesions [65]. Following a study on 270 male broiler chicks, Ledoux *et al*. [95] concluded that feed containing >50 mg/kg MON will exert toxic effects on these animals. The concentrations observed in this study are well below this limit. In fact, occurrence data summarised in the report submitted to EFSA suggest that naturally contaminated samples do not normally exhibit contamination at such a high level. Studies reporting MON concentrations near or exceeding this proposed tolerance level usually refer to contamination in handpicked, visibly *Fusarium*-damaged samples [93]. 

## 4. Experimental Section

### 4.1. Samples

From 2010 on, a total number of 83 naturally contaminated samples of feed and feed raw materials were analysed with a semi-quantitative multi-mycotoxin LC-MS/MS method. The majority of the samples were feed samples (*n* = 35). Besides, samples of silage (*n* = 21), maize (*n* = 15), wheat and wheat by-products (*n* = 6), barley straw (*n* = 3) and other feed ingredients (soybeans, sunflower seeds *etc.*, *n* = 3) were tested. The majority of the samples were sourced in Europe (*n* = 71), most often originating from Hungary (*n* = 19), Austria (*n* = 17) and Denmark (*n* = 15). Other European samples were obtained in Belgium, Italy, Norway, Russia, the UK and Ukraine. The remainder of the samples was sourced in America (*n* = 8, from Brazil, Canada and the USA) and Australia (*n* = 4). The pool of samples comprised a small number of samples (*n* = 9) that were known or expected to contain high levels of mycotoxins. These were three samples of US maize that had been found to contain high levels of DON (*n* = 2) or AF and FB (*n* = 1) using “conventional” HPLC analysis and six samples of maize cobs displaying different degrees of visible mould infection. The maize cob samples were all taken from the same field in Lower Austria in the 2010 (*n* = 2) and 2012 (*n* = 4) growing season.

### 4.2. Analysis

The analyses were conducted at IFA-Tulln. All samples were analysed for the presence and concentrations of fungal metabolites by LC-MS/MS according to Vishwanath *et al*. [8]. The analytical method has been extended to cover 320 metabolites, transferred to a more sensitive mass spectrometer and pre-validated for four matrices [96]. In brief, 5 g of sample was weighed into a 50 mL polypropylene tube (Sarstedt, Nürnbrecht, Germany) and extracted with 20 mL acetonitrile/water/acetic acid (79:20:1, *v*/*v*/*v*) for 90 min on a GFL 3017 rotary shaker (GFL, Burgwedel, Germany). Extracts were diluted in extraction solvent (ratio 1:1) and directly injected into the LC-MS/MS instrument. 

Chromatographic separation was performed at 25 °C on a Gemini^®^ C_18_-column, 150 × 4.6 mm i.d., 5 μm particle size, equipped with a C_18_ 4 × 3 mm i.d. security guard cartridge (all from Phenomenex, Torrence, CA, USA) and coupled to an 1290 Series HPLC System (Agilent, Waldbronn, Germany). Chromatographic separation was performed at 25 °C on a Gemini^®^ C18 column (Phenomenex, Torrance, CA, USA). After an initial time of 2 min at 100% eluent A, the proportion of eluent B was increased linearly to 50% within 2–5 min and 100% within 5–14 min, followed by a holding-time of 4 min at 100% eluent B and 2.5 min column re-equilibration at 100% eluent A pumped at a flow rate of 1 mL/min. 

A QTrap 5500 LC-MS/MS System (Applied Biosystems, Foster City, CA, USA) equipped with a TurboIonSpray electrospray ionization (ESI) source was used to detect and quantify the fungal metabolites. ESI-MS/MS was performed in the scheduled multiple reaction monitoring (sMRM) mode, both in positive and negative polarities in two separate chromatographic runs per sample by scanning two fragmentation reactions per analyte with the following settings: source temperature 550 °C; curtain gas 30 psi; ion source gas 1 (sheath gas) 80 psi, ion source gas 2 (drying gas) 80 psi, ion spray voltage −4500 V and +5500 V, respectively, collision gas (nitrogen) medium. The MRM detection windows were 54 and 96 s in the positive and negative ionization mode, respectively, and the cycle time was set to one second.

Mycotoxins were quantified by external calibration (1/*x* weighted) using a multi-component standard prepared from authentic standards.

## 5. Conclusions

Multi-mycotoxin LC-MS/MS analysis is a valuable tool for obtaining a more accurate picture of mycotoxin contamination pattern in feed and feed raw materials. Key advantages of such methods are the detection of masked mycotoxins and the detection of mycotoxins that are usually not tested for. 

Mycotoxin conjugates can make up a significant proportion of the total mycotoxin contamination, particularly regarding DON and ZEN [27]. In view of the possible reconversion of some mycotoxin conjugates (e.g., DON-3-Glc and ZEN-4-sulfate) to the parent toxin during digestion the occurrence of high proportions of masked mycotoxins might add significantly to the toxicity of the feed. Yet, little is known about the extent of masked mycotoxin reconversion during digestion (recent results suggest low bioavailability in rats [28]), and more research is needed in this field. 

The total number of detected metabolites amounts to 139, which is about twice as high as in previous studies applying the same method to African food and feed samples [21,22]. Yet, evaluating the risk associated with the presence of a given analyte proves difficult for the vast majority of the metabolites. Table 3 highlights the lack of toxicity data on these substances and the need for further research in this area. As exposure to acutely toxic concentrations is less common than chronic exposure to low levels of a metabolite, potential adverse effects of long-term low-level exposure should be the focus of future research efforts. 

The co-occurrence of seven to 69 metabolites per sample further complicates the evaluation of the feed material’s toxicological potential. Additive and synergistic effects on overall toxicity are frequently observed when mycotoxin mixtures are evaluated instead of single toxins. This is particularly true at sub-acute concentrations considering that most studies investigating the effect of mycotoxin combinations on animal performance at these levels found additive or synergistic effects [97]. In addition, naturally DON contaminated feed frequently proved more toxic than artificially contaminated feed containing the same concentration of the toxin [98,99]. This testifies to the fact that interactions of co-occurring metabolites can enhance the overall toxic effect of single mycotoxins. Consequently, data on the interactions of “novel” mycotoxins and other metabolites with each other and with regulated mycotoxins (AF, FB, DON, ZEN and OTA) are needed.

In conclusion, multi-mycotoxin LC-MS/MS analysis is an important step towards gaining a more accurate picture of the extent of mycotoxin contamination in agricultural commodities, food and feed. However, highlighting the frequent occurrence of both masked and usually neglected “emerging” mycotoxins, the results of such analyses also emphasise the fact that considerable research effort is still required in this field. In order to permit an accurate assessment of the risks associated with the presence of these contaminants more data is needed on their metabolic fate (particularly concerning masked mycotoxins), their toxicities and possible synergistic interactions with other mycotoxins present.
